# An integrative method to predict signalling perturbations for cellular transitions

**DOI:** 10.1093/nar/gkz232

**Published:** 2019-04-05

**Authors:** Gaia Zaffaroni, Satoshi Okawa, Manuel Morales-Ruiz, Antonio del Sol

**Affiliations:** 1 Luxembourg Centre for Systems Biomedicine (LCSB), University of Luxembourg, Esch-sur-Alzette L-4362, Luxembourg; 2 Integrated BioBank of Luxembourg, Dudelange L-3555, Luxembourg; 3 Biochemistry and Molecular Genetics Department-Hospital Clínic of Barcelona, Institut d’Investigacions Biomèdiques August Pi i Sunyer (IDIBAPS), Barcelona 08036, Spain; 4 Centro de Investigación Biomédica en Red de Enfermedades Hepáticas y Digestivas (CIBERehd), Barcelona 08036, Spain; 5 Working group for the biochemical assessment of hepatic disease-SEQC^ML^, Barcelona 08036, Spain; 6 Department of Biomedicine-Biochemistry Unit, School of Medicine-University of Barcelona, Barcelona 08036, Spain; 7 CIC bioGUNE, Bizkaia Technology Park, Derio 48160, Spain; 8 IKERBASQUE, Basque Foundation for Science, Bilbao 48013, Spain

## Abstract

Induction of specific cellular transitions is of clinical importance, as it allows to revert disease cellular phenotype, or induce cellular reprogramming and differentiation for regenerative medicine. Signalling is a convenient way to accomplish such transitions without transfer of genetic material. Here we present the first general computational method that systematically predicts signalling molecules, whose perturbations induce desired cellular transitions. This probabilistic method integrates gene regulatory networks (GRNs) with manually-curated signalling pathways obtained from MetaCore from Clarivate Analytics, to model how signalling cues are received and processed in the GRN. The method was applied to 219 cellular transition examples, including cell type transitions, and overall correctly predicted experimentally validated signalling molecules, consistently outperforming other well-established approaches, such as differential gene expression and pathway enrichment analyses. Further, we validated our method predictions in the case of rat cirrhotic liver, and identified the activation of angiopoietins receptor Tie2 as a potential target for reverting the disease phenotype. Experimental results indicated that this perturbation induced desired changes in the gene expression of key TFs involved in fibrosis and angiogenesis. Importantly, this method only requires gene expression data of the initial and desired cell states, and therefore is suited for the discovery of signalling interventions for disease treatments and cellular therapies.

## INTRODUCTION

Cellular phenotypes can be characterized by stable gene expression profiles maintained by the underlying gene regulatory networks (GRNs). Conversions between different cellular phenotypes (i.e. cellular transitions) can be induced either by perturbing directly GRNs, or cellular signalling pathways that in turn act on GRNs. These transitions range from cell type conversion events (reprogramming, differentiation), to conversions between cellular phenotypes within a cell type, due to drug treatment or disease conditions. The induction of desired cellular transitions is of clinical interest, as it allows to revert cellular disease phenotypes to their healthy counterparts, or to derive required cells and tissues for cell replacement therapies. By doing so without transfer of genetic material, but rather acting on signalling pathways, the safety concerns currently posed by gene therapy protocols can be overcome (1).

In order to systematically identify signalling perturbations induced by small molecules that are able to trigger changes in cellular phenotype, the effect of signalling on gene expression must be modeled. In this regard, two broad classes of computational methods have been recently developed, namely GRN-free and GRN-based approaches. Some of the GRN-free methods compare a signature gene list from the query gene expression profile with a compendium of signatures associated with known perturbations (2–4). Another set of GRN-free methods maps gene expression onto signalling pathways, and identifies the pathways or sub-pathways whose activities are (dys)regulated by using enrichment measures (5,6). Although these methods have been applied to identify signalling pathways perturbations inducing gene expression changes, they lack the mechanistic understanding of how a change in TF expression or activity gives rise to a new cellular phenotype. Thus, to capture how signalling can induce cellular transitions, a model that integrates signalling and gene regulatory networks is required. Existing GRN-based methods use ordinary differential equations (ODEs) to model the expression level of each gene as a function of the expression of its regulators (7–9). Nevertheless, ODE-based modelling frameworks cannot be applied to systems where only a small number of transcriptomics samples is available, as they require a large amount of data for parameter estimation. Various studies have presented manually-curated models where signalling and gene regulatory networks have been integrated to study individual cellular transitions (10–12). However, we are not aware of any computational approach that systematically integrates and models the signalling and transcriptional regulatory layers without requiring a large amount of gene expression data.

Here, we introduce a computational method that predicts signalling molecules whose perturbation can induce transitions between cellular phenotypes, given their initial and target gene expression profiles. This approach integrates the signalling network with a Boolean transition-specific GRN model. A central role in this model is played by interface TFs, which connect the two regulatory layers. The signalling information transmitted along pathways results in the activation or inhibition of interface TFs, which then act on the GRN, triggering cellular transitions. In line with previous observations (13,14), the transmission of signalling information is modeled as a probabilistic event depending on protein availability. Indeed, preliminary results show that the pathways predicted with this approach were enriched in proteins differentially phosphorylated upon perturbation, supporting their involvement in the signal transduction. Further, signalling molecules were ranked according to how effectively they act on the interface TFs that can induce the desired cellular phenotype. Importantly, by considering changes in the GRN initial state upon interface TFs perturbations, this method is able to model how signalling cues are received and processed in the GRN to give rise to the desired phenotype. To our knowledge, this mechanistic insight is not provided by other general methods.

We applied our method to 219 cellular transition examples, including the induction of cellular differentiation and reprogramming, and obtained correct predictions in the majority of them. Importantly, this method showed better performance than well-established GRN-free methods, and similar performance to another GRN-based method (DeMAND), while requiring substantially less data. Finally, the method was applied to the prediction of signalling molecules for the reversion of cirrhotic liver to its healthy counterpart. Experimental results confirmed that the activation of one of the predicted candidates restored the healthy expression state of key TFs involved in cirrhosis.

In summary, here we propose the first general method, to our knowledge, which uses gene expression data to identify signalling molecules able to induce cellular transitions. The low data requirements make this tool readily applicable to the design of new experimental protocols and the discovery of signalling perturbations for disease treatment or cellular therapies.

## MATERIALS AND METHODS

### Perturbation targets

Mapping of drugs and small molecules to their direct protein targets was carried out using STITCH (http://stitch.embl.de/ (15), v5.0, accessed in October 2017, with experimental evidence confidence >0.4); DrugBank (www.drugbank.ca (16), accessed in October 2017) and MetaCore from Clarivate Analytics were used to specify the effect (activation, inhibition, unknown effect). For growth factors and proteins, the interacting proteins were obtained from STITCH (same selection criteria), and the signalling network retrieved from MetaCore was used to define the effect on the targets.

### Datasets

All datasets contained in the Connectivity Map (build 02, (17)) and generated on Affymetrix Human Genome U133A 2.0 Array were processed. We also manually selected from ArrayExpress microarray experiments where expression data was collected before and after the application of a single perturbation, prioritizing non-cancer cell lines and, in particular, experiments related to cell differentiation or reprogramming. Datasets were discarded if all the targets of the used perturbation were either absent from the signalling network, or not connected to interface TFs by a directed path. In addition, experiments with chemically undefined perturbations (e.g. serum, oxygen, co-culturing conditions etc.) were also discarded. The evaluation was restricted to datasets with selective perturbation, meaning the number of target signalling molecules present in the signalling network was ≤30. These criteria allowed us to test our method on the prediction of well-characterized and specific signalling perturbations. The considered datasets contain expression values before and a few hours after the perturbation (6 h after drug application for CMap, and up to 48 h after the last perturbation in the manually selected datasets). In reprogramming examples, gene expression data from primary cells for both the initial and the desired cellular states was used.

### Phosphoproteomics datasets

Studies where a single perturbation was applied and quantified through phosphoproteomics data were paired to gene expression datasets matching the initial and final conditions as closely as possible. Ideally, the same cell type was perturbed with the same chemical, and gene expression was measured with comparable delay after perturbation. When this was not possible, time after perturbation was allowed to change up to 48 h. In addition, we considered closely related cell lines and different chemical compounds targeting the same protein targets. Regarding quantitative phosphoproteomics data, the list of differentially phosphorylated proteins were obtained from the original papers; when not available, we repeated the analysis as described in them (see Table 1). We used the highest fold change observed for any phosphosite on a protein as the fold change of that whole protein.

**Table 1.** Datasets for which both phosphorylation and gene expression data is available. Gene expression datasets are indicated with their GEO accession number

					Gene expression datasets (GEO access ID)	
Dataset	Cell type	Perturbation	Phosphorylation study (PMID)	LFC cutoff for DP	control	treated
Gnad *et al.*, 2016	HCT116 cells	MAPK inhibition [GDC0973 (1uM)]	27273156	log2(3)	GSM455560	GSM455565
Wilkes *et al.*, 2015	MCF7 cells	EGFR inhibition [EGFR2]	26060313	1	GSM149914	GSM149941
Rudolph *et al.*, 2016	MCF7 cells	EGF	28009266	2.38	GSM325937	GSM325958
						GSM325959
Sharma *et al.*, 2014	HeLa cells	EGF	25159151	1	GSM156764	GSM156770
D’Souza *et al.*, 2014	HaCaT cells	TGFβ	25056879	1	GSM297456	GSM297458
Wierer *et al.*, 2013	MCF7 cells	estradiol	23770244	log2(1.5)	GSM289651	GSM289654
					GSM289652	GSM289655
					GSM289653	GSM289656

The same log fold change values used in the original studies were used to define differential phosphorylation.

### Prior knowledge networks

#### Signalling network

We retrieved 75 canonical signalling pathways present in MetaCore from Clarivate Analytics in July 2017, and merged them together in a single signalling network, composed of 2496 nodes and 6876 edges. In MetaCore, all edges are obtained by expert manual curation of full text papers from literature, directed and signed when possible; the nodes represent signalling entities, either single proteins or complexes that act as functional entities. We removed edges corresponding to ‘Technical’ or ‘Unspecified’ effect, and ‘Technical’, ’Transcriptional Regulation’, ‘Influence on Expression’, ‘Catalysis’ and ‘Transport’ mechanisms. Some TFs are known to act as complexes but are represented in MetaCore as separate functional nodes all interacting with the same targets. We checked the literature supporting all the interactions involving these TFs, and manually removed the ones where the interaction did not specifically involve the TF considered, but other components of the complex (see Supplementary Table S1 for list of pathways used and the list of manually removed edges).

#### Transcriptional regulatory interactions

For transcriptional regulatory interactions, we considered all interactions among human TFs and transcriptional regulators as listed in Animal TFDB 2.0 (18), that are labelled in MetaCore as ‘Transcriptional regulation’, ‘Influence on Expression’ and ‘Regulation’, with ‘Activation’, ‘Inhibition’ or ‘Unspecified’ effect (accessed in March 2017).

### Microarray data processing

All data was processed starting from raw CEL files with the same pipeline, consisting of normalization with frozen-RMA (R package fRMA (19)) and assignment of expression state by Gene Expression Barcode (20,21). Briefly, the barcode approach assumes that the distribution of normalized, log-transformed expression values for a specific probeset observed across multiple tissues, cell types and conditions, can be fitted with a mixture model of a Gaussian distribution corresponding to non-expressed values, and a uniform distribution corresponding to expressed values (20). We selected for each gene the probeset with highest variance, then given this model, we assigned Boolean state 1 (expressed) to TFs that had probability lower than 0.05 of belonging to the non-expressed distribution (corresponding to parameter *cutoff* = 0.95 in the pipeline), and state 0 otherwise. Furthermore, in this work we defined the expression probability of a probeset $x$ as the ratio:

\begin{equation*}p\ \left( x \right) = \frac{{\frac{1}{2}{f_{e\left( x \right)}}}}{{\frac{1}{2}{f_{e\left( x \right)}} + \frac{1}{2}{f_{n\left( x \right)}}}}\ \end{equation*}

where ${f_e}$ is the probability density function (pdf) of $x$ in the uniform distribution $U( {\mu ,15} )$, and ${f_n}$ is the pdf of $x$ in $N( {\mu ,\sigma } )$ (see Supplementary Information and Supplementary Figure S1). To each protein, we assigned the maximum probability of expression found across all replicates. The values $\mu$ and $\sigma$ were calculated and distributed in (21) and are available as R Bioconductor packages for microarray platforms Affymetrix Human Genome U133A, Affymetrix Human Genome U133 Plus 2.0, Affymetrix Human Genome U133A 2.0, Affymetrix Human Gene 1.0 ST Array, Affymetrix Mouse Gene 1.0 ST Array and Affymetrix Mouse Genome 430 2.0 Array. While relying on pre-processed expression value distributions limits the application of our method to these specific microarray platforms, it also reduces the requirement for data samples to one sample per cellular state, one for the initial and one for the required state.

### Gene regulatory network (GRN) inference

TFs that were assigned different Boolean state (1 = expressed, 0 = not expressed) before and after the perturbation are assumed to also have differential activity in the two cellular states. These TFs were connected in a gene regulatory prior-knowledge network. This Boolean network was pruned so that the resulting GRN matches the initial and final Booleanized gene expression states, as described in (22). Briefly, this algorithm assumes that both states are represented by a separate point attractor in the Boolean network state space, and removes edges from the initial network in order to make the attractor states compatible with the Booleanized gene expression profiles. Datasets with GRNs containing <10 connected TFs after the pruning were excluded from further analysis, as we observed that they tend to correspond to perturbations affecting biological processes different from signalling (e.g. metabolic reactions, structural proteins).

### Addition of TFs connecting signalling pathways and GRN

The activity of signalling molecules on the GRN is mediated by the TFs that belong to signalling pathways and therefore can act as signalling effectors (*interface TFs*). TFs that are not expressed initially will require some time to be expressed, and are not likely to be involved in the signalling response. Additionally, we assumed that whatever signal is applied to the cell, it initially travels to the nucleus using proteins that are already expressed in the cell. Therefore, in this study we defined *interface TFs* as TFs that regulate TFs present in the GRN (*GRN-TFs*), are expressed at the initial time point, and are connected through expressed signalling paths to any of the source nodes (0-indegree nodes) of canonical signalling pathways. No further filtering was applied (Supplementary Figure S2).

### *In silico* perturbations of interface TFs

The interface TFs that are more likely to drive the cells from the initial to the final gene expression profile were found by *in silico* perturbations of the initial GRN state. We exhaustively tested combinations of up to four interface TFs at the same time, by fixing their Boolean state and updating synchronously the Boolean state of the network following a majority (threshold) logic rule until it converged to a fixed-point attractor. Interface TFs have the property of being directly regulated by signalling events, meaning that their expression at the initial cellular state is not sufficient to assume they are also active or inactive. Unless an interface TF had different Boolean states between the initial and final gene expression, its activity state at the initial time point was unknown. Therefore, we simulated the network state by assigning it both 0 and 1 states.

The perturbations are ranked according to their flipping score, which is the number of GRN-TFs that change their state after simulation. We then selected the combinations of interface TFs that obtained the three best (including ties) flipping scores (best performing combinations, BPCs). This corresponds to parameter *best* = 3 in the pipeline. We removed the combinations of interface TFs that did not show any synergistic effects (i.e., combinations whose flipping TFs were the same as the union of flipping TFs of individual constituent interface TFs). This is because our method aims to prioritize candidate signalling molecules that specifically target interface TFs over those that target a large number of interface TFs, in order to avoid unspecific effects on cells upon their perturbations. Prioritizing interface TFs that synergistically maximize the flipping score allows us to select BPCs with fewer, but more specific, interface TFs that need to be targeted by optimal signalling perturbations (Supplementary Figure S3). Datasets for which the best flipping score did not represent at least 40% of the GRN-TFs were discarded from further analysis.

### Probability of intermediate signalling molecules regulating interface TFs

We approximated the regulation of signalling molecule *x* on interface TF *y* by considering the most probably expressed path (MPP) connecting *x* to *y*. The probability *M_x,y_* of the MPP is defined as:

\begin{equation*}\ {M_{x,y}} = \ p \left({MP{P_{x \to y}}} \right) = \ max\ \ p\left( {x \to \ldots \to y} \right)\end{equation*}

where $p\ ( {x \to \ldots \to y} ) = \mathop \prod \nolimits_{j \in proteins \in simple\ path} p( j )$ and *p*(*j*) is the expression probability of the intermediate signalling molecule *j*. We used two variations of this approach (see Supplementary Information and Supplementary Figure S4):

- Proteins belonging to the same functional modules tend to show transcriptional correlation (23). Accordingly, we calculated the Pearson correlation coefficient of expression values across all the processed datasets in CMap, and we increased the probability of interactions among proteins that were correlated at the expression level (absolute correlation > 0.7 and sign of correlation matching the sign of the interaction). We used these corrected probabilities to identify the MPPs and calculate their correlation-corrected probability.
- We expect that the longer the path used to reach an interface TF (measured in number *n* of interactions present in the path), the higher the chances that crosstalk and non-functional interactions occur. Therefore, the probability of the MPP connecting between each signalling protein and interface TF was multiplied by ${e^{ - n}}$ (24), resulting in what we defined as length-corrected probability.

The distribution of probabilities ***P_x_*** from the same signalling protein *x* to all interface TFs was obtained by:

\begin{equation*}\ {P_{x,y}} = \frac{{{M_{x,y}}}}{{\mathop \sum \nolimits_{i \in interfaceTFs} {M_{x,i}}}}\ \end{equation*}

Two ***P_x_*** vectors exist for each molecule, one corresponding to correlation-corrected probability, and one length-corrected probability. Finally, we determined the sign of each MPP by

\begin{equation*}{sign_{x,y}} = \mathop \prod \nolimits_{e = edges \in MPP} sign\left( e \right)\ \end{equation*}

If the sign is positive, the activation (inhibition) of *x* causes the activation (inhibition) of TF *y* with probability *P_x,y_*. If the sign is negative, activation (inhibition) of *x* causes inhibition (activation) of *y* with probability *P_x,y_*.

### Prediction of candidate signalling proteins for gene expression state transitions

We assumed that a high frequency of a particular activated/inhibited interface TF among the BPCs is a good indication that it consistently has large effects on the GRN state. For each state assigned to each interface TF (TFs-state pair *s*) the frequency *F_s_* in all BPCs was calculated as:

\begin{equation*}\ {F_s} = \frac{{{k_s}}}{k}\ \end{equation*}

where *k* is the total number of BPCs and *k_s_* is the number of combinations where *s* is present. The frequencies across all TF-state pairs *S* were then normalized to sum up to one, giving the probability distribution ***Q***:

\begin{equation*}Q\ = \frac{{{F_i}}}{{\mathop \sum \nolimits_{i \in S} {F_i}}}\ \end{equation*}

The ranking of the signalling molecules was obtained by comparing their probability of reaching the interface TFs (***P_x_***) with the frequency of such TFs in the BPCs (***Q***) by Jensen-Shannon divergence:

\begin{equation*}JSD \left( {{P_x}\parallel Q} \right) = \frac{1}{2}\ D\left( {{P_x}\parallel M} \right) + \frac{1}{2}D\left( {Q\parallel M} \right)\end{equation*}

where $M\ = \frac{1}{2}\ ( {{P_x} + Q} )$ and $D\ ( {X\parallel Y} ) = \mathop \sum \nolimits_i X( i )log\frac{{X( i )}}{{Y( i )}}$ (Kullback–Leibler divergence). Both activation and inhibition of the signalling protein *x* are possible, but their scores differ because the sign of the paths connecting *x* to the interface TFs will be opposite, resulting in different effects on the GRN state. For example, assume that the activation of *x* results in the activation of TF *y*, which is frequently present in the BPCs. The inhibition of *x* on the other hand, assigns to *y* the inactive state, which is not present in the BPCs. Here the activation of *x* would have a better score than its inhibition, because while the probability of reaching TF *y* is the same, the resulting perturbations on the GRN have different effectiveness in changing its state.

The signalling molecules were ranked by JSD values (the smaller the better), and then assigned a rank:

\begin{equation*}R \left( x \right) = \mathop {min}\limits_{v \in {P_x}} \ rank\left( {x,v} \right)\end{equation*}

where *R*(*x*) is the rank assigned to signalling molecule *x*, and is defined as the minimum rank obtained by *x* using either correlation- or length-based ***P_x_*** variant (*v*).

A cut-off was defined as a fraction (from 1 to 10%) of the maximum *R* value present in the final ranking. The signalling molecules whose rank R was lower than the cut-off were considered candidate drivers of the transition between initial and final states. For single perturbation datasets, the prediction was considered successful if at least one of the direct targets of the experimental perturbation appeared among the candidates, as was also done in (25). At each cut-off, the chance of obtaining at least one perturbation target (i.e. a success) in a randomly selected set of the same size was calculated by one-sided hypergeometric test. The optimal cut-off was selected as the one where our method showed the maximum improvement from the random chance, across the datasets coming from CMap.

### Functional analysis

GO biological process terms enrichment was calculated separately for candidate and non-candidate signalling molecules using the R package *gProfilerR*. The overlap between GO terms associated to experimental perturbation targets (target terms) and enriched terms was counted for each dataset and the distributions of these values were compared by one-sided Wilcoxon test.

The distances on the signalling network topology were calculated from all signalling molecules to experimental perturbation targets, and in the opposite direction. The direction with shorter distance was then selected, and the average distance from one signalling molecule to all the reachable experimental perturbation targets was calculated. The distributions of average distances from candidate and non-candidate signalling molecules were compared by Wilcoxon test with 100 000 Monte Carlo replicates (*P*-value < 0.05).

### Experimental model of cirrhosis and CVX-060 treatment

To induce cirrhosis, 10 male Wistar rats were exposed to inhalation of CCl_4_, as previously described (26) and according to the criteria of the investigation and ethics committee of the Hospital Clínic Universitari and the University of Barcelona. Five cirrhotic rats were treated once a week with 10 mg/kg of CVX-060 (Pfizer, Inc., New York, NY, USA) for 4 weeks. CVX-060 was diluted in 500 μl of saline solution and injected intravenously via the tail vein.

### Prediction of signalling molecules for reversion of cirrhotic state

Expression data for healthy liver in male Wistar rats was obtained from GEO dataset GSE71201. Gene expression in CCl_4_ and CCl_4_+CVX-060 treated livers was quantified using Affymetrix GeneChip Rat Genome 230.2 Array. After quality control and PCA visualization, two replicates for each treatment were retained for further analysis. Each gene was assigned gene expression probability equal to $1 - p$, where *p* is the *P*-value obtained from Affymetrix MAS5.0 detection call. A gene was considered expressed if its expression probability was equal or larger than 0.94 (corresponding to call ‘marginal’ or ‘present’ from MAS5.0).

The prediction of GRN state after CVX-060 treatment was obtained by first selecting all the interface TFs in the BPCs that are activated/inhibited with probability higher than zero by the activation of Tie2. Then, the BPCs composed only of such TFs are selected, and the GRN-TFs that change their state in any of these BPCs are expected to flip when CVX-060 is applied. The same GRN state is also obtained when predicting the effect of the inhibition of Ang2 on the GRN state.

### Comparison with previously published methods

#### Differential gene expression

Differential gene expression between the initial and query expression profiles was calculated with the R package *limma*. Genes with absolute log fold change (lfc) >log_2_(1.5) and BH-adjusted *P*-value <0.05 were considered differentially expressed. When replicates were not available, the lfc cut-off alone was applied. MetaCore pathway enrichment was calculated by one-sided hypergeometric test (BH-adjusted *P*-value < 0.05). To rank all signalling molecules according to differential expression, they were ordered by decreasing absolute lfc values.

#### SPIA

Differentially expressed genes (DEGs) were selected by BH-adjusted *P*-value <0.1, or by lfc >log_2_(1.5) if replicates were not available. The R package *SPIA* (27) was used to calculate KEGG signalling pathways significantly perturbed. A prediction was considered successful if any of the significant pathways contained any of the direct targets of the perturbation applied in a dataset. Datasets without any DEG were discarded.

#### Connectivity Map

DEGs were selected by BH-adjusted *P*-value <0.05, or by lfc >log_2_(1.5) if replicates were not available. Up to 150 up- and down-regulated genes, by decreasing absolute lfc, were submitted to the ‘Batch query’ functionality of CMap L1000 query, accessible at https://clue.io/l1000-query#batch (28), with sig_fastgutc_tool option enabled. The summary results across cell lines were used for further analysis. The predictions were considered correct if the experimental perturbation, its direct targets, or a drug targeting the perturbation targets were assigned a connectivity score (*tau*) >90. Datasets with <10 DEGs, or raising errors during the submission, were discarded from the analysis.

#### DeMAND

Our method was applied to the GEO datasets used as part of the benchmarking for DeMAND (termed GEO13 in the original paper)(29). Only datasets generated on Affymetrix whole-transcriptome array platforms were considered. When available, values $\mu$ and $\sigma$ (21) were used to calculate the probability of expression; otherwise, the Affymetrix MAS5.0 detection call was used as previously described. Perturbation targets from STITCH, DrugBank, as well as the original paper, were used to define successful predictions. In DeMAND, genes with FDR≤.1 were considered predicted candidates.

## RESULTS

### Method overview

We present here a computational method that predicts signalling molecules, including plasma membrane receptors or intermediate signalling proteins, whose perturbations can induce desired cellular transitions. It requires only gene expression profiles of the initial and desired cellular states, and does not require a large number of replicates, time-series expression profiling, or phosphoproteomics data. Therefore, this method can be applied to the transition between any pair of initial and query cell states, including novel cellular transitions that have not been achieved previously.

**Figure 1.**
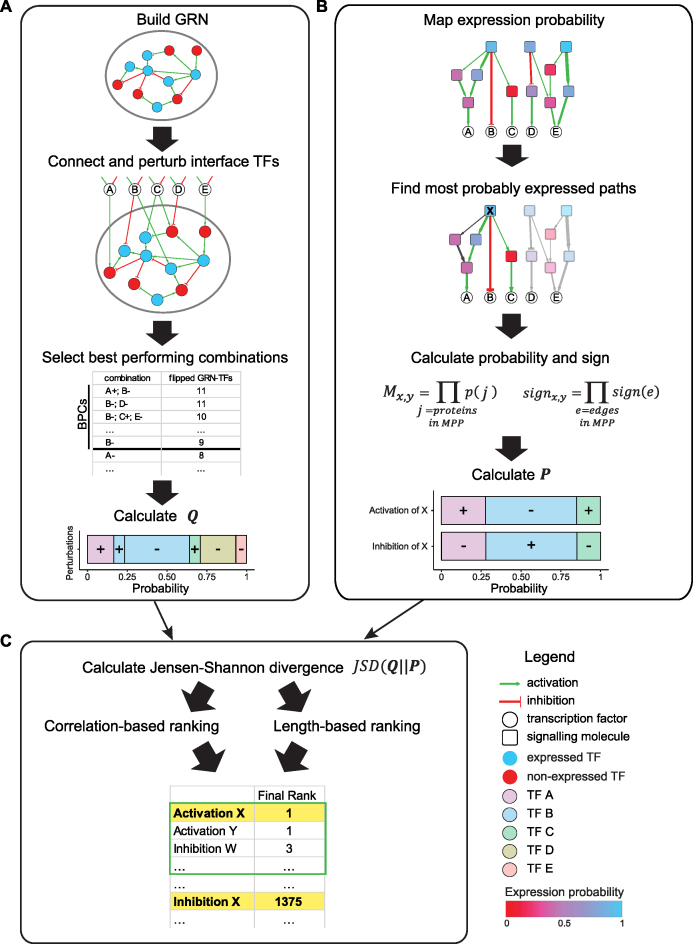
Ranking signalling molecules according to their likelihood of inducing desired changes in GRN state. (**A**) The gene regulatory network (GRN) containing differentially Booleanized TFs is connected to interface TFs. The initial state is perturbed by in silico simulation of fixed states of up to four interface TFs at the same time. The best performing combinations (BPCs) are selected as the ones having the top three flipping scores (including ties). The frequency of each interface TF state (activated +, inhibited –) is calculated and then normalized to give the probability distribution Q of each TF state of causing changes in the GRN state. (**B**) The expression probability of each protein is mapped onto the signalling network and used to define the probability of signalling interactions. For each signalling molecule X the most probably expressed paths (MPPs) connecting it with all interface TFs are selected. The probability and the sign of the MPPs are calculated, and combined to give the probability distribution P of activating or inhibiting the interface TFs by activating or inhibiting X. Both correlation-based and length-based probabilities are calculated (see Methods). (**C**) The probability distributions Q and P are compared through Jensen-Shannon divergence (JSD). The score is used to rank the perturbations of each signalling molecule. The best ranking that each molecule obtains across the correlation-based and length-based rankings defines its final rank. A fraction of the final ranking is selected.

In the first step of the pipeline, the activity of TFs is approximated by Booleanizing their expression state. TFs with differential activity are selected and connected to form a Boolean transition-specific GRN. In this framework, the cellular states are modelled as network state attractors resulting from the same network topology. The objective is to induce the transition from the initial to the desired attractor by acting on TFs that are regulated by signalling interactions (i.e. interface TFs). Perturbations of these interface TFs are performed exhaustively to identify the ones that are most effective for the GRN state transition (Figure 1A).

In the next step, we predict the effect of the activation/inhibition of each molecule in the signalling network on the downstream interface TFs. Signal transduction is an inherently stochastic process, strongly dependent on post-translational protein modifications not captured by gene expression data (30). However, it has been shown that the response to signalling perturbations changes across different cell types depending on the abundance of specific signalling proteins prior to perturbation (13,14). Thus, we model signal transduction as a probabilistic process driven by protein availability, which can be estimated from gene expression data, an approach followed also by other methods (31,32). We define the probability of signal transduction from a signalling molecule to interface TFs as the product of the expression probability of all proteins present in the MPP between them. Two variants of the signalling probability are used: (a) the probability of an interaction is higher if the genes involved are correlated in gene expression, since their proteins are more likely to work as a functional unit (23); (b) the probability of a path is multiplied by the exponential of its length, to account for the number of interactions required for the signal to reach the TFs (i.e. the longer, the less probable). The sign of each MPP is then incorporated into its outcome, i.e. if the overall sign of a path is positive, the activation/inhibition of that path will activate/inhibit the target interface TF, whereas if the overall sign is negative, the activation/inhibition of that path will inhibit/activate the target TF (Figure 1B).

Finally, our method ranks each signalling molecule in the network based on the Jensen-Shannon divergence of the probability *P*, with which it acts on interface TFs, from the likelihood *Q* of the interface TFs themselves of changing the GRN state (Figure 1C).

### Phosphoproteomics datasets suggest that MPPs are used in signal transduction

While not all proteins are subject to phosphorylation, many signalling pathways rely on phosphorylation cascades for transmitting the signal in the cytoplasm. Therefore, phosphoproteomics experiments allow the inference of protein activity during a signalling event (33). Proteins showing differential phosphorylation upon perturbation are expected to be transmitting the newly applied signal, and therefore the signalling paths used for signal transduction should show an enrichment in differentially phosphorylated proteins compared to the paths that are not used for signal transduction. Given this assumption, we asked if the MPPs selected by our method were enriched with phosphorylation events.

**Figure 2.**
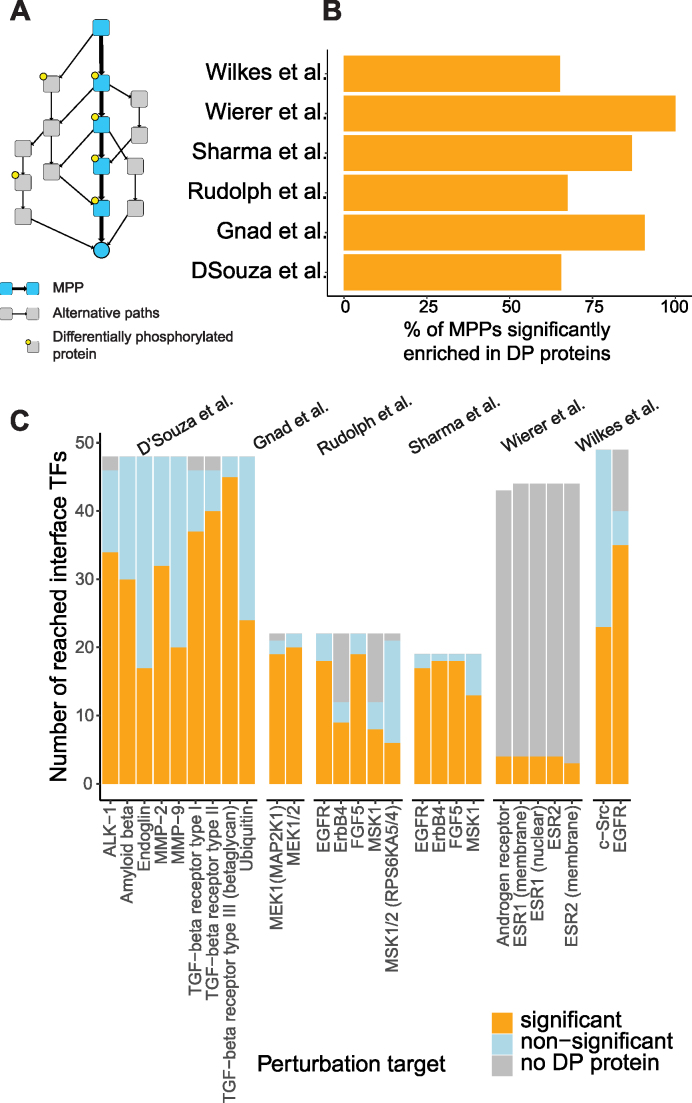
Enrichment of MPPs in differentially phosphorylated (DP) proteins. (**A**) MPPs are defined by correlation-based and length-based probabilities. For both of these methods, the fraction of DP proteins in each MPP for each target-interface TF pair is compared by t-test to other simple paths connecting the same pair. (**B**) Average fraction of MPPs per datasets significantly enriched in DP proteins compared to alternative simple paths containing DP proteins (*P*-value < 0.05). (**C**) Breakdown of the single MPPs. Orange: number of interface TFs for which the fraction of DP proteins in the MPP is significantly higher than in other simple paths. Light-blue: the difference is not significant; grey: there are no DP proteins in any of the paths connecting the perturbation target to interface TFs. The same results were obtained with both correlation-based and length-based MPPs.

To investigate this, we gathered experiments for which both gene expression and quantitative phosphoproteomics data were acquired before and after a specific perturbation was applied (Table 1). In each of the datasets, the direct target molecules of the experimental perturbation and the interface TFs that they can affect via signalling were identified, and the MPP between each pair of perturbation target molecule and interface TF was computed (Figure 2A). While a protein might contain multiple phosphosites, not all of them are necessarily functional. However, at the moment, only a limited number of proteins have their phosphosites functionally characterized, and for the majority of the proteins this information is not available (33). Therefore, we first considered a protein differentially phosphorylated (DP) if any of its phosphosites showed differential phosphorylation, defined in the original studies (see Table 1). We then calculated the frequency of DP proteins in the MPP, and in randomly selected simple paths among the same initial and final nodes (up to 100 randomly selected paths, limited to maximum path length = 10 edges, Figure 2A). The difference between the frequency of DP proteins in the MPP and the random paths was tested for statistical significance (*t*-test, *P*-value < 0.05). In each dataset, the majority of the MPPs had significantly more DP proteins than other possible paths, for both probability computation methods we used to define MPPs (Figure 2B and C). Therefore, the MPPs between signalling molecules and interface TFs reasonably captured phosphorylation patterns observed shortly after signalling perturbations. This gave us confidence in using MPPs as biologically relevant paths used by signal transduction.

### Prediction of signalling molecules that induce desired gene expression change

We applied our method to datasets belonging to CMap generated on Affymetrix Human Genome U133A 2.0 Array, plus datasets manually selected from ArrayExpress. After quality controls, 219 datasets (193 from CMap, 26 from ArrayExpress) were used for the analysis. For each dataset, a GRN was built and perturbed *in silico* to obtain BPCs. General characteristics of GRNs and BPCs are summarized in Supplementary Information and Supplementary Figure S5. In order to generate the final signalling molecule ranking, a) the probability with which each signalling molecule can act on interface TFs, and b) the likelihood of the interface TFs to induce the desired GRN state transitions, are compared using Jensen-Shannon divergence (see also Methods). Signalling molecules that specifically reach a few well-performing interface TFs will score better than molecules which indistinctly act on many interface TFs. On average, ∼1400–1500 signalling molecules are present twice in the final ranking, once for their activation and once for their inhibition, resulting in approximately 2900–3000 potential perturbations for each dataset.

**Figure 3.**
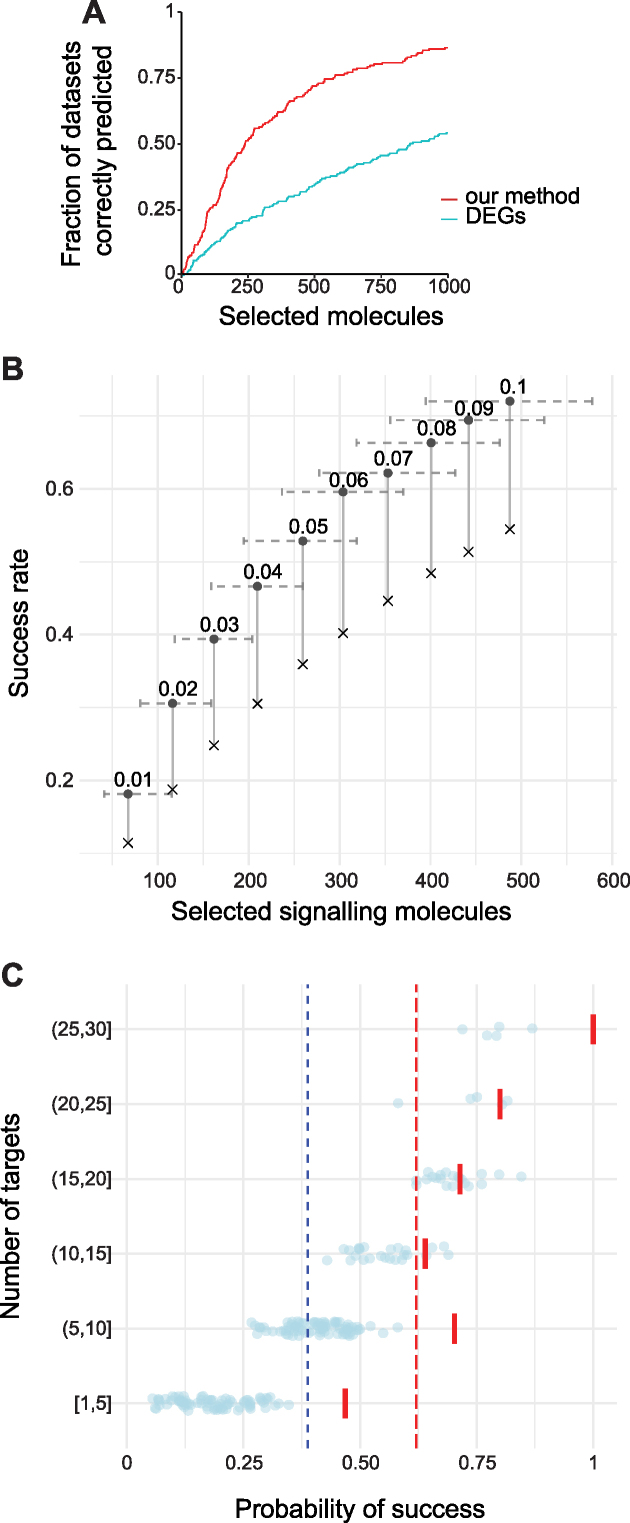
Method performance. (**A**) Fraction of datasets where at least one target is correctly predicted, across an increasing selection of signalling molecules. Proteins are ranked according to their expression log fold change between the initial and final gene expression profile (DEG), or according to our method. (**B**) Variation of success rate and number of selected signalling molecules, at different ranking cut-offs. Circles = observed success rate, X = random success rate, horizontal error bars = 5th and 95th percentiles of selected set size. The same fraction of ranks selected corresponds to variable set sizes because of ties in the ranking. Sets size range was removed from the random success rate points for clarity. (**C**) Performance across datasets with increasing number of known perturbation targets. Each point represents the random success rate for a dataset, obtained by calculating how likely it is to find at least one perturbation target in a random set of signalling molecules of same size as the one selected by our method. The solid red lines represent the fraction of datasets in each class, for which at least one of the targets was in the list of candidate signalling molecules selected by our method. Red dashed line: average obtained performance across all classes (62%), blue dashed line: random average performance (39%).

First, we compared the ranking obtained with our method with one based on differential gene expression. Ordering genes by their log fold change did not prioritize perturbation targets, which were found only after selecting a big portion of the ranking, while our method performed better (see Figure 3A). For example, to retain a correct experimental perturbation in at least 50% of the datasets, 858 molecules need to be selected by log fold change, compared to the 239 required by our method. This confirms that proteins involved in signal transduction do not necessarily show differential expression, and therefore more complex approaches are needed in order to obtain better predictions using gene expression data.

We considered a prediction correct if at least one of the known perturbation targets appeared in the top ranked molecules. The success rate was calculated as the fraction of datasets for which the prediction was correct. Different ranking cut-offs were tested, and for all of them, the success rate on CMap datasets was better than the random selection of the same number of candidates (Figure 3B). Cut-off = 0.06 was used for subsequent analysis because our method showed the highest performance gain at this cut-off with respect to the random success rate, with 136 out of the total 219 datasets (62%) being successful (Supplementary Table S2). In particular, it correctly predicted at least one perturbation target in 115/193 CMap examples (60%, versus random success of 41%, Figure 3B and C), 6/10 datasets for non-cancer cell lines selected from ArrayExpress, 5/6 datasets with matched phosphoproteomics data (Table 1), and 10/10 cell type transitions datasets (discussed below). We observed that our method is particularly successful in predicting signalling molecules for cellular transitions where a higher number of differentially expressed genes exists between the initial and desired cellular states. This suggests that acting on the GRN with signalling perturbations can be an effective strategy, especially for cellular transitions requiring broad changes in gene expression (Supplementary Figure S6).

The number of direct targets of a compound or protein influences the probability of finding at least one of them among the selected candidates. Therefore, we divided the datasets in different classes based on the number of targets reported for the corresponding perturbation. For each of these classes, the performance of our method was compared to the frequency with which at least one real target is expected to appear in random sets of signalling proteins of the same size (Figure 3C). A significantly better performance was obtained in datasets with 1–10 known perturbation targets (*P*-value = 7.82e–05 for datasets with 1–5 targets, and 3.40e–06 for 6–10), which represent 74% of all datasets tested. The use of target-specific drugs or growth factors is required to induce cellular transitions in a controlled way, and these results demonstrate that our method is particularly suited for such cases. As the majority of the datasets analysed with our method concerned drug application, but so few of the drug–target pairs had a known sign (16% of all pairs), we could not comprehensively assess the accuracy of the predicted signs. However, we did not observe a bias in our method towards the prediction of inhibition or activation of signalling molecules (Supplementary Figure S7).

There exist multiple other drug-perturbation gene expression datasets, for example the data generated for the DREAM/NCI compound synergy challenge (34), which are often used to benchmark methods that use gene expression data to predict cellular response to drugs (9,29). However, we could not estimate the gene expression probability for those datasets because their microarray platforms are incompatible with our method.

### Properties of candidate signalling molecules

**Figure 4.**
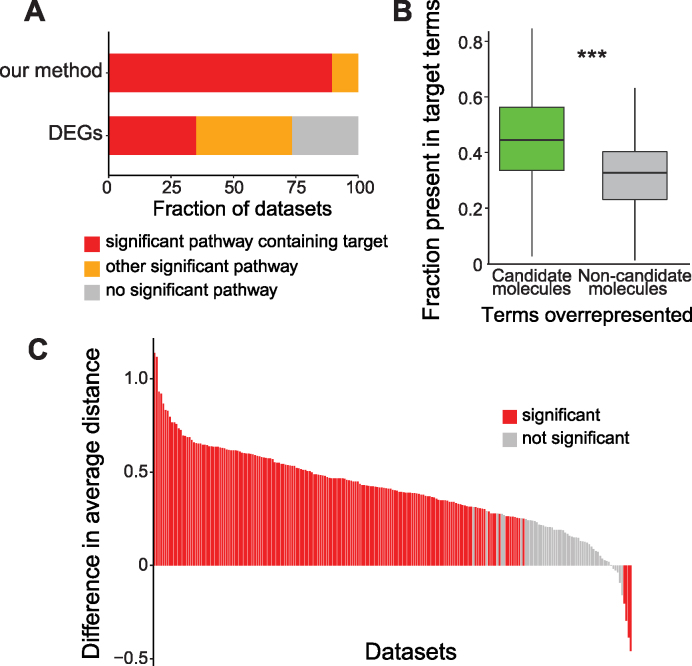
Features of candidate molecules vs. other molecules. (**A**) Fraction of datasets for which the enrichment of pathways in signalling molecules selected either by our method or through differential expression, finds a pathway containing perturbation targets (red), any other pathway (orange), or no pathway significantly enriched. (**B**) Percentage of functional terms mapping to the perturbation targets also enriched in the selected molecule set, or the discarded molecule set. Whiskers indicate 1.5 * inter-quantile range. The candidates have significant higher portion of enriched functional terms shared with the perturbation targets (one-sided Wilcoxon test, *P*-value = 6e–16, confidence interval = (0.1048031,Inf)). (**C**) The average distance from perturbation targets of candidates is smaller than the average distance of non-candidates. Red bars = significant difference of the distance distributions, grey bars = non-significant difference (Wilcoxon test with 100 000 Monte Carlo replicates).

Next, we asked whether the sets of predicted candidate signalling molecules were related to direct perturbation targets by analysing their functional and topological features. First, the canonical signalling pathways extracted from MetaCore were tested for overrepresentation among candidate signalling molecules. We observed the enrichment of at least one pathway in all datasets. Moreover, 89% of the times at least one of the pathways containing known targets was enriched (Figure 4A). To further evaluate this result, we also tested enrichment of MetaCore canonical pathways in DEGs. 73.5% of the datasets showed some pathway enrichment, but only in 35.2% of all datasets at least one of the enriched pathways contained perturbation targets (Figure 4A). This result indicates that also at signalling pathway level, our method is far more effective in predicting appropriate signalling perturbations than simply using DEGs. In addition, we collected all the GO biological process terms associated with the perturbation targets (target terms). Then, we calculated which GO terms were enriched in the candidate signalling molecules and in the non-candidate signalling molecules. The target terms were more frequently overrepresented in the candidate signalling molecules than in the non-candidate ones (*P*-value = 6e–16, Figure 4B).

To investigate where the candidate signalling molecules were located in the signalling network, we compared the distribution of distances (minimum number of interactions with same direction required to connect two nodes) from selected candidate molecules to perturbation targets, and from non-candidate molecules to targets. We found that the distances were overall significantly shorter for candidate molecules in 72% of the datasets, significantly longer in 2% of the datasets, and comparable in 26% (Figure 4C). This suggests that our method selects molecules that are not randomly scattered in the signalling network, but are found in the region where the applied perturbation acts.

In summary, candidate signalling molecules are more involved in the same biological processes as the perturbation targets than non-candidate molecules or DEGs. Additionally, they are distributed in the signalling network in proximity to perturbation targets. These results suggest that candidate signalling molecules are likely to induce the desired cell state transition, and are novel candidates for further experimental validation.

### Application to cell type transitions

In the context of regenerative medicine, the ability to induce cellular conversions between different cell types would allow to replace damaged tissues and organs. We tested our methods on datasets where growth factors or drugs were used to alter the cellular identity. These cases showed larger GRNs compared to the CMap datasets (on average 37.5 versus 23 TFs), and overall better performance: in all datasets the candidate signalling molecules contained direct targets of the experimental perturbation (Table 2), compared to the 60% success rate obtained across CMap datasets.

**Table 2.** Results obtained on cell type transition examples. Ranks passing the 6% cut-off, which correspond to correct prediction, are reported in bold. Type: D = differentiation, A = activation, M = maintenance, R = reprogramming. Cell types: MSC = mesenchymal stromal cells from the bone marrow; HSPC = hematopoietic stem/progenitor cells; NHEK = normal human epidermal keratinocytes; HME = hematopoietic microenvironment in bone marrow

Type	Initial cell type	Perturbation	Final cell type	Ref.	Best rank	Predicted direct targets	notes
D	hMSC	BMP2	chondrocytes	(35)	10	ALK-2	TGF-β3 targets predicted: TGF-beta receptor type III (betaglycan)
						Chordin__inh	
						BMP receptor 2	
						Noggin__inh	
						Ectodin__inh	
D		TGF-β3			38	Endoglin__inh	BMP2 targets predicted : Chordin | Noggin | Ectodin | PTCH1__inh
D	HSPC	valproic acid	Erythroid and megakaryocytic precursors	(36)	5	HDAC9__inh	
						HDAC2	
D	NHEK	density-induced differentiation, treated with EGF	terminally differentiated keratinocytes	(37)	1	ErbB4	
						MSK1	
D	hepatoblasts	cAMP	hepatocyte-like cells	(38)	143	Protein kinase G1	
A	pre-adipocytes	dexamethasone	primed pre-adipocytes	(39)	93	DAX1	
A	mesenchymal stem cells	bFGF	non-HME cells	(40)	24	Casein kinase II, alpha chains	
						Casein kinase II, alpha' chain (CSNK2A2)	
A		TGF-β1	subendothelial mural cell fate		103	Ubiquitin	bFGF targets predicted: Syndecan-3__inh | Casein kinase II, alpha' chain (CSNK2A2)__inh | S100B__inh
M	hES-T3	activin A + bFGF	-	(41)	86	ALK-4	Protocols comparison: MEF feeder
M			-		15	ALK-4	Protocols comparison: feeder-free
						ALK-7	
						ALK-2__inh	
R	Mouse embryonic fibroblasts	CHIR99021 + RepSox + Forskolin + valproic acid	cardiomyocytes	(42)	166	RepSox: JNK1(MAPK8)__inh	
R	Adult fibroblasts	SP600125 + SB202190 + Go6983	hMSC	(43)	89	SP600125: p38beta (MAPK11)__inh; JAK3__inh; MSK1__inh	Go6983: cPKC (conventional) (opposite sign)
						SB202190: p38beta (MAPK11)__inh; p38alpha (MAPK14)__inh	

#### Differentiation

In (35) the differentiation of human mesenchymal stromal cells to chondrocytes was obtained by treatment with either BMP2 or TGF-β3. Comparing the gene expression profiles of the BMP2-treated cells to the untreated ones, we predicted that the activation of both BMP receptor 2 and TGFBR3 would induce the differentiation, in accordance with the experimental evidence. The activation or inhibition of other members of the TGF-β protein superfamily, which is known to play an important role in chondrocyte differentiation, was also predicted when using both target gene expression profiles. We also correctly predicted the activation of HDAC2 and the inhibition of HDAC9 for the differentiation of hematopoietic stem/progenitor cells to erythroid and megakaryocytic precursors (36); the application of EGF during differentiation of neonatal keratinocytes to terminally differentiated keratinocytes (37); and the activation of protein kinase G1 (PRKG1), a direct interactor of cAMP, as inducing hepatoblasts differentiation towards a hepatocyte-like population (38).

#### Cell activation and maintenance

The activation of pre-adipocytes to primed pre-adipocytes can be obtained with dexamethasone treatment (39), and in agreement with this observation we predicted the activation of DAX1, a nuclear receptor for steroid hormones. Mesenchymal stem cells can give rise to many cell types with different potential to establish a hematopoietic differentiation microenvironment. This particular competency is inhibited by treatment with bFGF; the treatment with TGF-β1 on the other hand pushes the cells towards subendothelial murate cell fate (40). Applying our method to bFGF-treated data, we predicted the activation of subunits of the protein kinase CK2, known to bind and phosphorylate bFGF. When applied to TGF-β1-treated data, our method did not predict any direct target apart from ubiquitin, but it suggested the inhibition of bFGF targets. Regarding maintenance of stem cells, in (41) the authors compared MEF feeder and feeder-free protocols for maintenance of hESC-T3 in vitro, to treatment with activin A in conditioned medium. They observed that self-renewal and pluripotency are preserved, but the mRNA and miRNA expression profiles were significantly different for the cells maintained with activin A. When comparing the activin A treatment with the other protocols, our method correctly predicted the activation of ALK-4 (activin A type IB receptor), activation of ALK-7, and inhibition of ALK-2.

#### Reprogramming

Cellular reprogramming is increasingly obtained with chemical cocktails. We tested our method on the direct conversion of mouse fibroblasts into cardiomyocytes obtained in (42) with a combination of four compounds (CHIR99021, RepSox, Forskolin and valproic acid). Using data from primary cells, our method only predicted one direct target of RepSox. However, it predicted the activation of Axin, which is a common target of GSK3, a kinase that is inhibited by CHIR99021, and G-protein alpha-s, one of Forskolin targets. Valproic acid, still, did not have any target or target-first neighbor in the candidate signalling molecules. We also applied our method to the conversion of primary human dermal fibroblasts into mesenchymal stem cells. It was observed that the minimal combination of SP600125, SB202190 and Go6983 is sufficient to obtain MSC-like induced cells (43). Our method correctly captured three direct targets of SP600125 (the inhibition of p38, JAK3 and MSK1) and two SB202190 targets (the inhibition of p38 in its α and β forms). Go6983 is an inhibitor of protein kinases C, for which our method predicted instead the activation. This can arise from the fact that multiple equally probable paths with opposing signs can exist, but only one MPP is selected as representative of the effect of a signalling molecule on an interface TFs.

In summary, our method consistently predicts signalling perturbations that can induce cell type transitions. In addition, it can predict alternative ways of obtaining the same cellular conversion, as observed in the differentiation of human mesenchymal stromal cells to chondrocytes, and mutually exclusive perturbations, as in the specification of subendothelial murate cell fate in mesenchymal cells. This confirms that not only experimentally perturbed targets are predicted, but also other selected signalling molecules are biologically relevant. No other computational method is known to be capable of systematically predict meaningful signalling molecules for the induction of cell type transitions.

### Application to disease treatment

**Figure 5.**
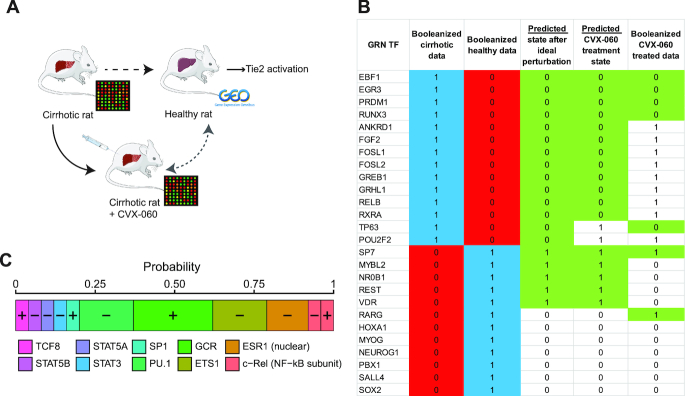
Application to cirrhotic model in rat. (**A**) Our method was applied to gene expression data of whole liver from cirrhotic and healthy rats, respectively generated in this study and obtained from public repositories. The activation of the angiopoietins receptor Tie2 was predicted as potential signalling perturbation able to convert the disease towards the healthy phenotype. Cirrhotic rats were treated with the specific Tie2 activator CVX-060, and gene expression was profiled again and compared with the data corresponding to healthy animals. (**B**) Boolean state of GRN TFs as measured in cirrhotic, healthy, and CVX-060 treated samples. The ideal perturbation state refers to the state that the GRN TF can reach if any of the BPCs is applied. The predicted CVX-060 treated state is the state the GRN-TFs can have if the BPCs composed only of interface TF states induced by the activation of Tie2, according to our predictions (using the correlation-based MPPs). Green background is used when a state is matching the desired healthy state. (**C**) Interface TFs present in the best *in silico* perturbations and their relative probability of inducing the desired changes on the GRN. +: the activation of the interface TFs acts on the GRN; –: its inhibition affects the GRN. The two states are not mutually exclusive, see c-Rel (NF-kB subunit).

Finally, we applied this method to the prediction of signalling molecules for disease treatment. In particular, we analysed cirrhotic versus healthy rat liver in order to induce the shift in the gene expression state of the diseased tissue towards the healthy state (Figure 5A). Currently, the therapeutic prospects for cirrhosis patients are limited to liver transplantation and, therefore, there is an urgent need to develop new therapeutic strategies. Cirrhosis was induced in male Wistar rats with CCl_4_ treatment, and RNA from the complete livers was extracted and quantified through microarray experiments. The gene expression profile of the desired healthy liver state was obtained from publicly available data (see Materials and Methods). The GRN built by our method consisted of 26 TFs (Figure 5B). After *in silico* perturbation of the 106 interface TFs connecting this GRN to the signalling network, we identified 10 TFs that were present in the BPCs (Figure 5C). Altogether, the BPCs were predicted to change the state of 19 GRN-TFs (Figure 5B).

The overall ranking of signalling molecules prioritized many proteins known to be involved in different aspects of liver fibrosis, fatty liver disease, cirrhosis, and hepatocellular carcinoma. In particular, the inhibition of fibrosis-related proteins was predicted (e.g. CHIP, AP-1, CBP, MDM2), along with the activation of ESR2, known for its antifibrogenic effects (44), and the inhibition of MMPs responsible for matrix remodelling. Multiple interleukins and proteins related to innate immune response in liver cirrhosis (45) were also selected as candidates. Another biological process emerging in our predictions is angiopoietins signalling, a key pathway in blood vessel normalization. Angiopoietin 1 (Ang1)-Tie2 signalling stabilizes blood vessels, Angiopoietin 2 (Ang2) on the other hand is a context-dependent antagonist of Ang1 and decreases its stabilizing effect (46) giving rise to immature blood vessels. Cirrhotic conditions are characterized by higher expression and activity of Ang2 than healthy conditions, resulting in the loss of blood vessels stability. In this regard, the activation of angiopoietins receptor Tie2 ranked 24th among all signalling molecules, and our method also predicted the activation of Angiopoietin 1 and 4 and the inhibition of Angiopoietin 2 and 3 (Supplementary Table S3). Our model predicted that the activation of Tie2 would activate the interface TFs SP1 and ETS1, and inhibit GCR, STAT5A and B, ESR1, and PU.1 (Supplementary Figure S8), inducing a GRN state that partially matches the healthy liver state (Figure 5B). To test this prediction, we generated gene expression data from the whole liver of cirrhotic rats treated with CVX-060, an inhibitor of Ang2 which induces Tie2 activation, and observed that the GRN-TFs EBF1, EGR3, PRDM1, RUNX3, SP7, RARG and TP63 were indeed reverted to the healthy state (Figure 5B). EGR3 and RUNX3 have been implicated in the pathophysiology of fibrosis and liver development. EGR3 is a pro-inflammatory and immunogenic factor, its overexpression is sufficient to stimulate fibrosis, whereas suppression of Egr-3 activity in deficient mice correlated with the attenuation of the TGF-β signalling and consequently of fibrogenesis (47). EGR3 is also an essential effector of VEGF-mediated functions leading to angiogenesis (48). Runx3 is mostly expressed in the liver during the embryonic development and is a regulator of fetal hematopoiesis (49). Runx3 knockout mice died within 24 h after birth showing organogenesis defects in lung and liver. In addition, the absence of Runx3 activity was associated with excessive intrahepatic angiogenesis, suggesting that the physiological function of this TF in the liver is mainly embryonic (50). RA signalling through RARG has been shown to reverse hepatic stellate cell activation and fibrosis (51), SP7 and TP63 have been previously implicated in the regulation of VEGF-mediated angiogenesis, while PRDM1 and EBF1 have no clear connection with angiogenesis or cirrhosis reported to this date and could be novel therapeutic targets.

Next, we constructed a GRN for the treated gene expression profile, and compared it with the disease GRN. The treated GRN had a similar size to the disease GRN (30 TFs), and shared 14 TFs with it. However, the 12 TFs that were exclusively present in the disease network were localized in network-specific modules that contained TFs playing a major role in vascular growth, including the disease-specific TFs TP63, EGR3, RUNX3 and SP7, discussed above (Supplementary Figure S8). On the other hand, modules shared between the disease- and the treated GRNs did not contain TFs associated with angiogenesis. This confirms that the activation of Tie2 specifically targeted the transcriptional regulators of angiogenesis, and reverted their gene expression state to the healthy counterpart.

Taken together, this experimental study provides insights into the molecular changes during the inhibition of Ang2 with CVX-060 in cirrhotic rat liver. Importantly, it complements the previous functional study where inhibition of Ang2 and activation of the Tie2 signalling have been demonstrated to improve the normalization of intrahepatic blood vessels and to decrease the liver inflammatory infiltrate, and thus an effective treatment for liver fibrosis in cirrhotic rats (52). As activation of Tie2 only partially reverts the disease phenotype, a combination of candidates involved in different aspects of the disease is probably necessary to obtain the complete switch towards a healthy expression profile. In this context, endothelin inhibition is another predicted candidate (ranking 17th overall, see Supplementary Table S3) that plays an important pathological role in cirrhotic livers through a different mechanism, fibrogenesis induction. Independent studies have convincingly described that an overexpression of endothelin-1 is associated with the pathological activation of hepatic stellate cells, which are the major source of collagen expression in the liver, and intrahepatic vascular dysfunction through exacerbated vasoconstriction (26).

### Comparison with existing methods

**Figure 6.**
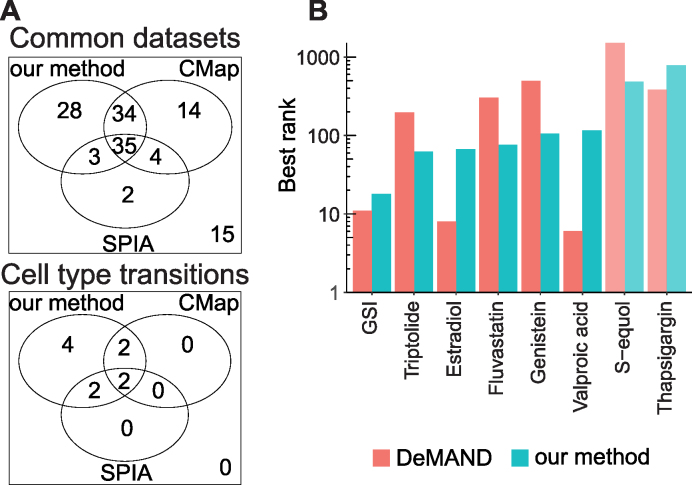
Comparison with previously published methods. (**A**) Performance of our method, Connectivity Map (CMap) and SPIA on datasets that could be analysed with the three methods (upper panel), and on cell type transition cases (lower panel). The number of cases in which the predictions of one or more methods were correct are reported. (**B**) Performance of DeMAND and our method on the eight compound perturbation datasets that could be analysed with both. Both methods correctly predicted direct perturbation targets for all datasets except (S)-equol and thapsigargin.

No method similar to ours in terms of application or modelling strategy exists to date. Therefore, we compared our method to computational tools that differ from ours by approach and application, but which are widely used to analyse signalling perturbations using gene expression data. Connectivity Map (28) uses gene expression similarity between a compendium of known perturbations and a query signature (list of up- and down-regulated genes) to rank small molecules, drugs and gene perturbations. SPIA (27) scores signalling pathways by their enrichment in DEGs, while also taking into account their topology. We applied both Connectivity Map and SPIA to the 219 datasets considered in our analysis, and obtained results for respectively 136 and 211 datasets where the tools could be applied and the prediction of the experimental perturbation was possible. We then focused on the 135 datasets that obtained predictions from all three methods (ours, Connectivity Map and SPIA). (Figure 6A, Supplementary Table S4). SPIA correctly predicted KEGG pathways containing direct perturbation targets in only 33% of such datasets. Connectivity Map in turn correctly predicted either the experimental perturbation, its gene targets, or drugs regulating the same targets, in 64% of the datasets, and our method obtained correct signalling molecules in 74% of the datasets. While Connectivity Map has an overall success rate similar to our method, it was not very successful in cell type transition datasets, only retrieving correct perturbations in four of them (Figure 6A). SPIA showed a performance similar to Connectivity Map in these cases. This result suggests a superior performance of our method in predicting novel perturbations, which are not present in the Connectivity Map compendium.

We also compared our method to DeMAND (29), a GRN-based tool aiming at identifying compounds mode of action using gene expression profiles and context-specific regulatory networks. Considering each gene G and the list of genes that it can regulate, called regulon, DeMAND scores each gene G based on how significantly the expression of its regulon is dysregulated following drug application. As it requires at least six samples for each condition to give reliable results, DeMAND could not be applied to the datasets analysed with our method, and we could not assess its suitability for the induction of cell fate transitions. Instead, we tested our method on the compound perturbation datasets that were used for DeMAND’s evaluation. We obtained candidates in nine datasets, however in one case the perturbation targets were absent from the signalling network used in our method, and therefore their prediction was not possible. Both our method and DeMAND obtained correct predictions in six of the remaining eight datasets (75%) (Figure 6B, Supplementary Table S4). Aside from obtaining comparable performance to DeMAND using substantially less data, our method explicitly predicts the activation or the inhibition of signalling molecules, and correctly reported signs in 5/6 datasets, thus overcoming an important limitation of DeMAND.

## DISCUSSION

Here we have introduced the first general method, to our knowledge, which uses gene expression data to predict signalling perturbations that can induce the transition from an initial to a desired cellular phenotype. For this purpose, single signalling molecules are prioritized according to their probability of specifically acting on the interface TFs that are most likely to trigger the shift from the initial to the required GRN state.

Our approach differs conceptually from previously published studies since it constitutes a general methodology that integrates signalling and gene regulatory networks by considering transitions between GRN states corresponding to the initial and target cellular phenotypes. On the contrary, other GRN-based approaches solely rely on GRN topology, and therefore ignore collective changes in TF expression induced by the signalling cues. Furthermore, our method was more successful than GRN-free methods in predicting signalling targets for cellular conversions, and showed similar performance compared to another GRN-based method (DeMAND), which requires higher amount of data. Importantly, the pathways predicted by this method to be involved in signal transduction were supported by changes in phosphorylation state (when data was available), indicating that gene expression alone can be reasonably used to analyse signalling processes in the absence of phosphoproteomics and perturbation data.

Results show that our method is able to consistently identify signalling targets of experimentally validated perturbations, and novel candidates with potential to induce desired cellular transitions. In particular, our method correctly predicted experimentally validated signalling targets in the analysed cell type transition examples, including cellular differentiation, reprogramming. Further, we applied our method to a liver cirrhosis model in rat to predict signalling molecules whose perturbations could revert the disease phenotype. Experimental perturbation of the predicted angiopoietins receptor (Tie2) induced desired changes in the gene expression of key TFs involved in fibrosis and angiogenesis.

An important limitation of our method is that it only predicts single signalling molecules, whereas combinations of these molecules could improve the efficiency of cellular conversion. In this regard, this method could be extended to the prediction of combinations of signalling molecules in order to take into account the combinatorial effect of multiple signalling molecules, which could be synergistic or redundant.

In conclusion, we believe that this method represents a general tool that can guide the identification of signalling molecules for the induction of desired cellular transitions, such as the reversal of disease phenotypes and the induction of cell differentiation or reprogramming, with perspective applications to disease treatment and regenerative medicine.

## DATA AVAILABILITY

The method was implemented in Matlab and R. It is available as a Snakemake pipeline (53) with all necessary datasets at: https://git-r3lab.uni.lu/gaia.zaffaroni/INCanTeSIMO. All microarray data generated is available in GEO under accession number GSE122822.

## Supplementary Material

gkz232_Supplemental_Files
