# Supplementary material for: An integrative method to predict signalling perturbations for cellular transitions
Source: Nucleic Acids Res. 2019 Apr 5;47(12):e72. doi: 10.1093/nar/gkz232 (PMC6614844; doi:10.1093/nar/gkz232)
Supplement: gkz232_Supplemental_Files [file gkz232_supplemental_files.zip › Supplementary table legends.pdf]

SUPPLEMENTARY MATERIAL LEGENDS:

Table S1. List of canonical signalling pathways extracted from MetaCore from Clarivate Analytics (accessed in July 2017), and list of interactions removed after manual curation, because the associated literature was not supporting direct interaction between the two proteins. Only interactions that involved TFs known to work in complexes which are not present as functional units in MetaCore, were manually checked.

Table S2. Dataset information and results for all datasets analysed.

Table S3. Predictions for cirrhotic to healthy liver conversion (rank 1-50).

Table S4. Results obtained with SPIA, Connectivity Map and DeMAND.
