# Supplementary material for: An integrative method to predict signalling perturbations for cellular transitions
Source: Nucleic Acids Res. 2019 Apr 5;47(12):e72. doi: 10.1093/nar/gkz232 (PMC6614844; doi:10.1093/nar/gkz232)
Supplement: gkz232_Supplemental_Files [file gkz232_supplemental_files.zip › SupplementaryInfo_revised.pdf]

SUPPLEMENTARY INFORMATION

Calculation of expression probability values

Intuitively, if the probability of a gene expression value belonging to the non-expressed distribution is 0, the $p(expression)$ should be 1. If there is some probability that the value could belong to the non-expressed distribution, then the $p(expression)$ should be lower. To capture this idea, we decided to define the $p(expression)$ corresponding to a gene expression value as the fraction of its total probability density coming from the expressed distribution. This ensures that $p(expression)$ ranges between 0 and 1 when a value might belong to both the expressed and not-expressed distribution, but reaches 0 when the expression value tested equals or is lower than the average value of the non-expressed distribution (the most frequent value, as the distribution is normal) (Supplementary Figure S1).

To model the expressed distribution, the uniform distribution was selected for practical purposes: it does not require further parameters which are not available, and still grants that as soon as a value does not belong to the non-expressed distribution, the gene will be assigned $p(expression) = 1$.

Calculation of the most probably expressed paths

We calculate the probability of a path as the product of the probabilities of the nodes used on it. An efficient algorithm to do this calculation is not available, but the summation along paths used to define shortest paths is a common problem in graph theory, and many algorithms and implementations are available.

We assigned to each edge *e* a weight w_e:

$$w_e = ln\frac{1}{p_{t_e}}$$

Where p_{t_e} is the probability of expression of the target of edge *e*. For the correlation-corrected MPPs, before calculating the shortest paths we divided by two the weights of the edges that connected two nodes correlated in gene expression (Supplementary Figure S4).

Summing these weights corresponds to multiplying the expression probability of the nodes. For this, we used the Dijkstra's shortest paths algorithm implementation present in the R package *igraph*. After obtaining the weight of the shortest paths, we transformed these weights back to probability values, to obtain the probability of a signalling molecule to reach an interface TF.

Identification of most efficient interface TFs to induce GRN state transitions

In the majority of the datasets considered, we observed a limited number of TFs responding to the perturbation by changing their expression state, and the size of the GRNs connecting them was moderate (on average 23 TFs, see Figure S5). The number of interface TFs connected to the GRN varied across datasets between 12 and 151, with an average of 38 TFs. Once in silico perturbations were applied, in each dataset the best perturbation flipped on average 70.8% of GRN-TFs. The perturbations obtaining the top three "flipping scores" (including ties) were selected as the best performing combinations (BPCs) of interface TFs for the subsequent

analyses. TFs that were present in less than 1% of the BPCs were labelled as rare. BPCs composed exclusively of rare TFs represent only 0.006% of the BPCs across all datasets, indicating that the BPCs are mostly composed of recurring interface TFs. It is not possible to enumerate all the combinations containing a given interface TF to define how likely it is to change the GRN state, but we assumed that the prevalence of recurring TFs would also appear in combinations of five and more interface TFs perturbed at once. Thus, we used the frequency of interface TFs in the BPCs as an approximation of their effectiveness in changing the GRN state across any perturbation size.

SUPPLEMENTARY FIGURES

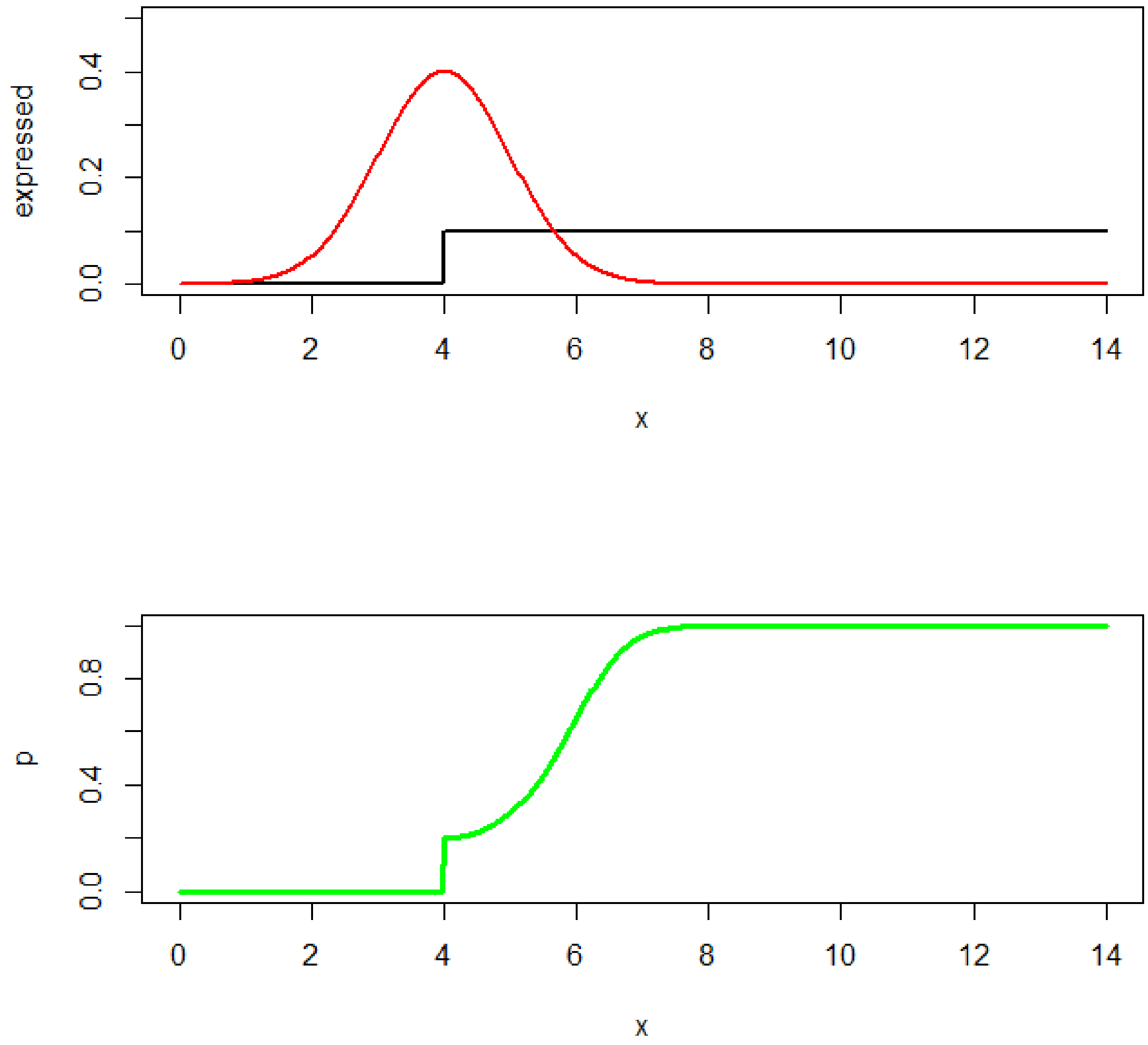

Figure S 1 A) Distributions used to calculate the expression probability: in red is the non-expressed distribution, a Gaussian distribution of known mean and standard deviation (obtained from fRMA packages); in black is the expressed distribution, which is a uniform distribution between the mean of the non-expressed distribution and 15, the maximum value fRMA-normalized expression levels can have. B) The form of the expression probability distribution.

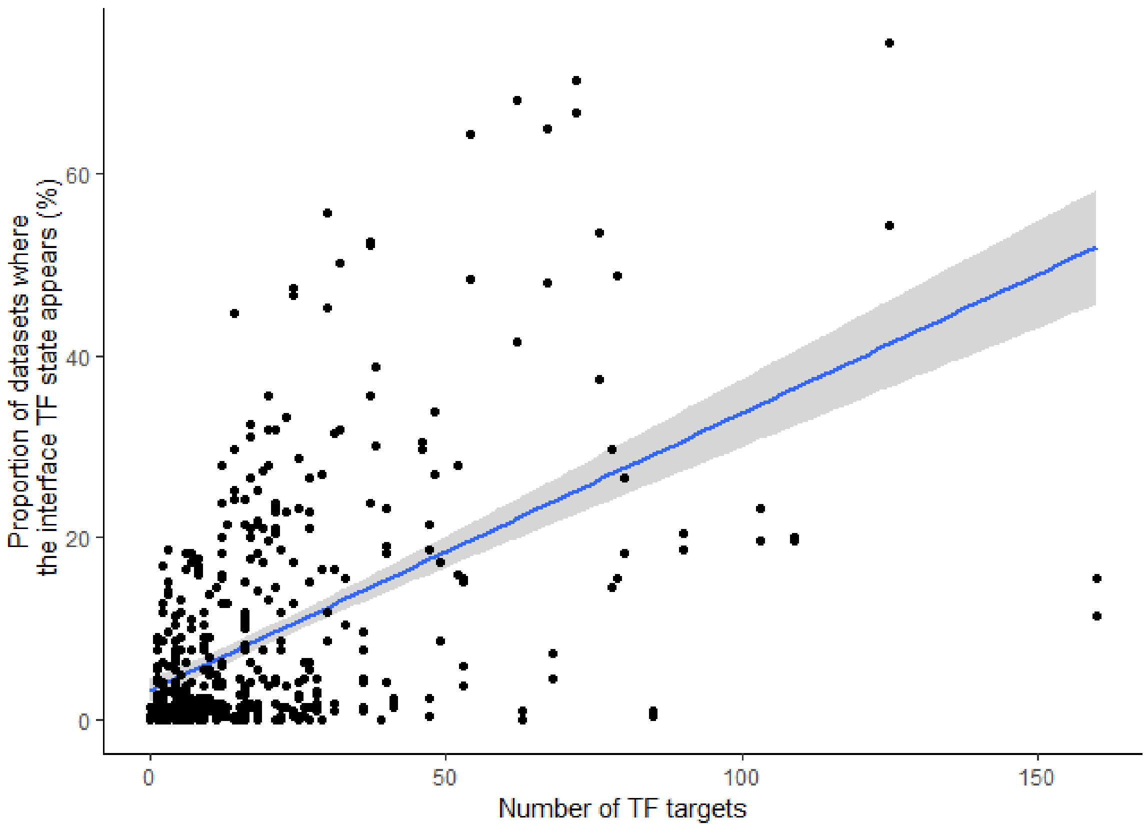

Figure S 2 Relation between the presence of an interface TF state in the BPCs of multiple datasets (y-axis), with the TF number of transcriptional GRN-TF targets (x-axis). The coefficient of determination R2 is moderate, indicating a limited influence of the number of targets of an interface TF with its recurrence in the BPCs of more datasets. 538 interface TF states are present across the 219 datasets considered.

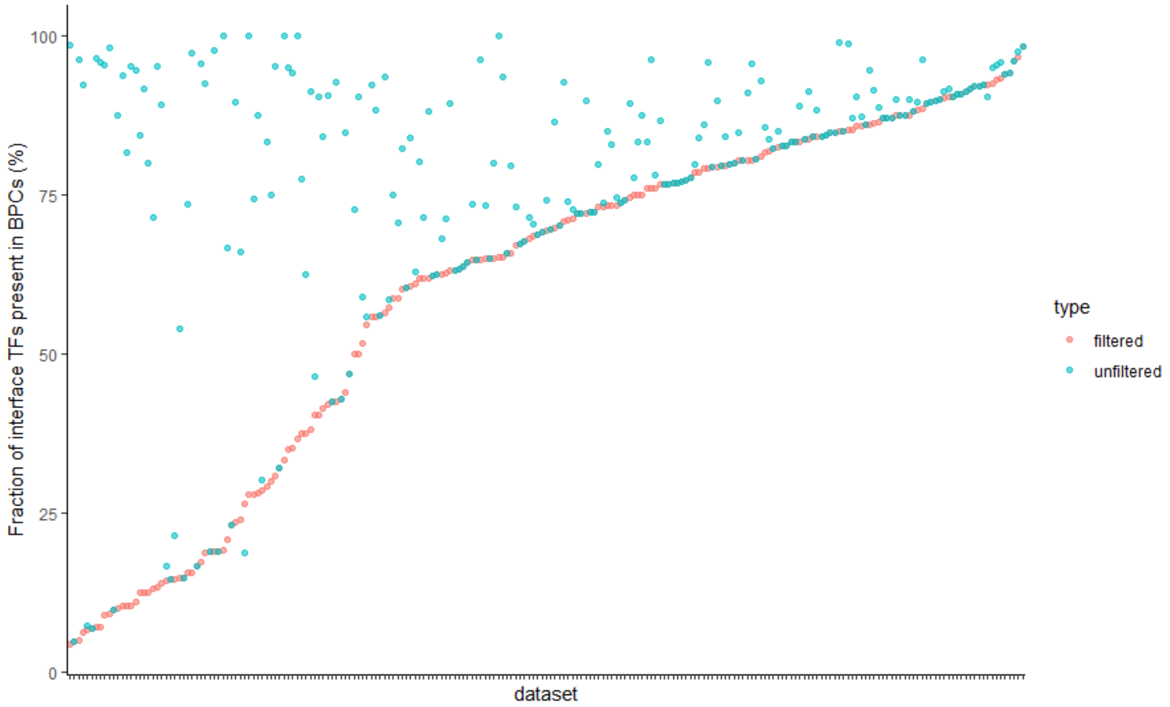

Figure S 3 Fraction of interface TFs present in the BPCs across all 219 datasets. BPCs are selected by a) simply considering all the combinations having the three best scores (“unfiltered”, blue); b) by considering the combinations with synergistic activity of the involved TFs, such that the combination is affecting the state of more GRN-TFs that the sum of its components (“filtered”, red). Apart from two cases, the unfiltered BPCs contain an equal or higher fraction of interface TFs compared to the filtered BPCs.

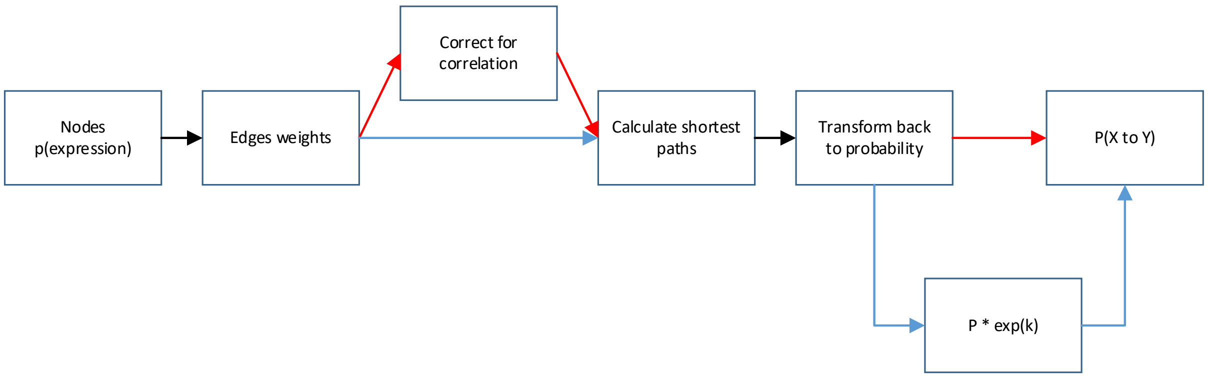

Figure S 4 Calculation of the probability of signalling molecule X to reach interface TF Y. Red: strategy used for correlation-corrected MPPs, blue: length-corrected MPPs, black: common steps.

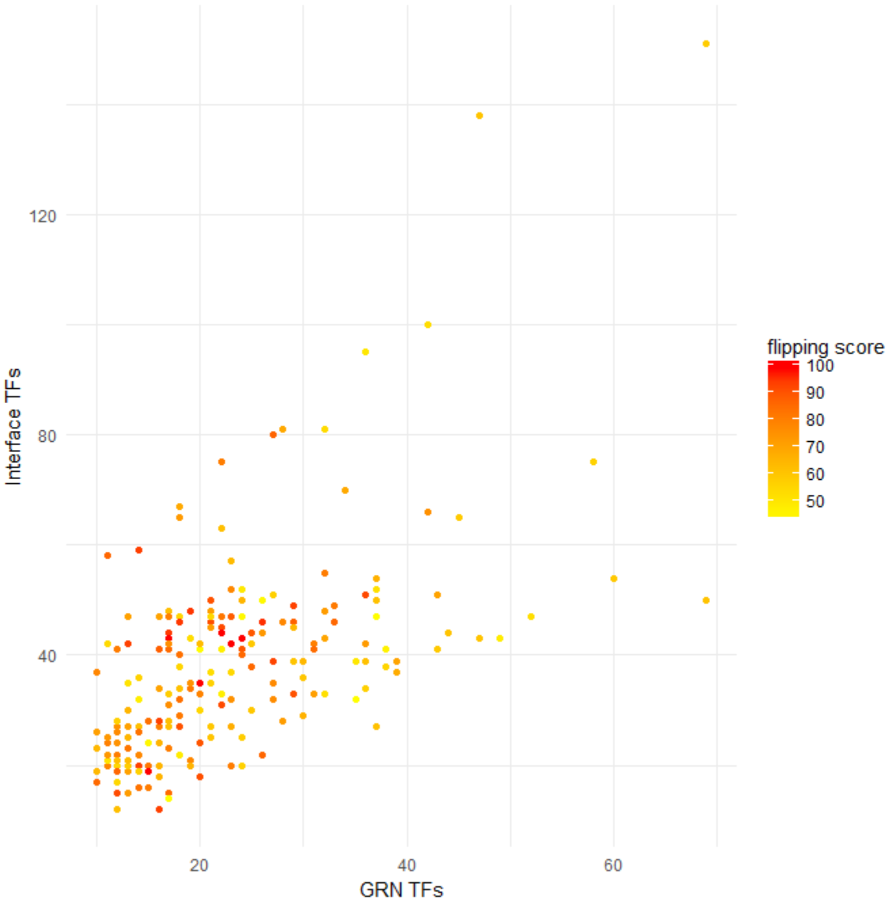

Figure S 5 Properties of integrated signalling-GRN models for all datasets considered. The size of the GRN and the number of the interface TFs is shown, together with the maximum flipping percentage (fraction of GRN TFs that change their state) obtained with in silico simulations.

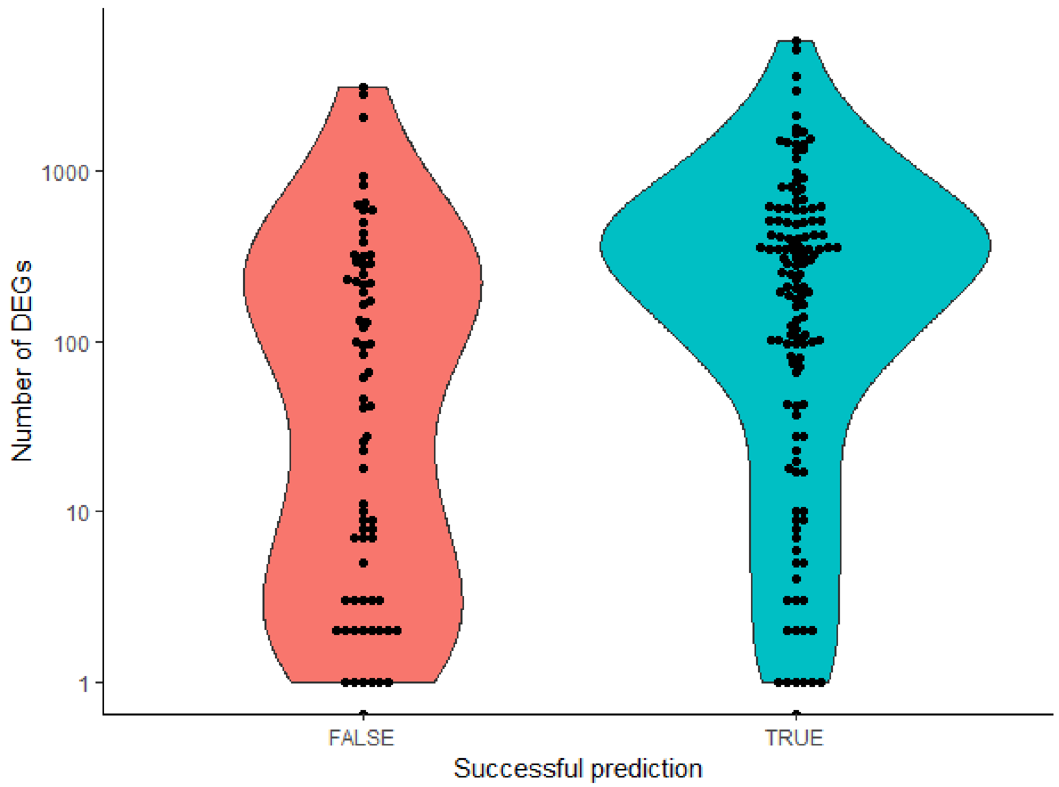

Figure S 6 Success of the predictions as a function of the difference between initial and final cellular states. The number of differentially expressed genes between the two gene expression profiles is significantly higher for the datasets where the prediction with our method was successful, than in the datasets where the predictions were not correct (one-sided Mann-Whitney test, p-value = 6.239e-07).

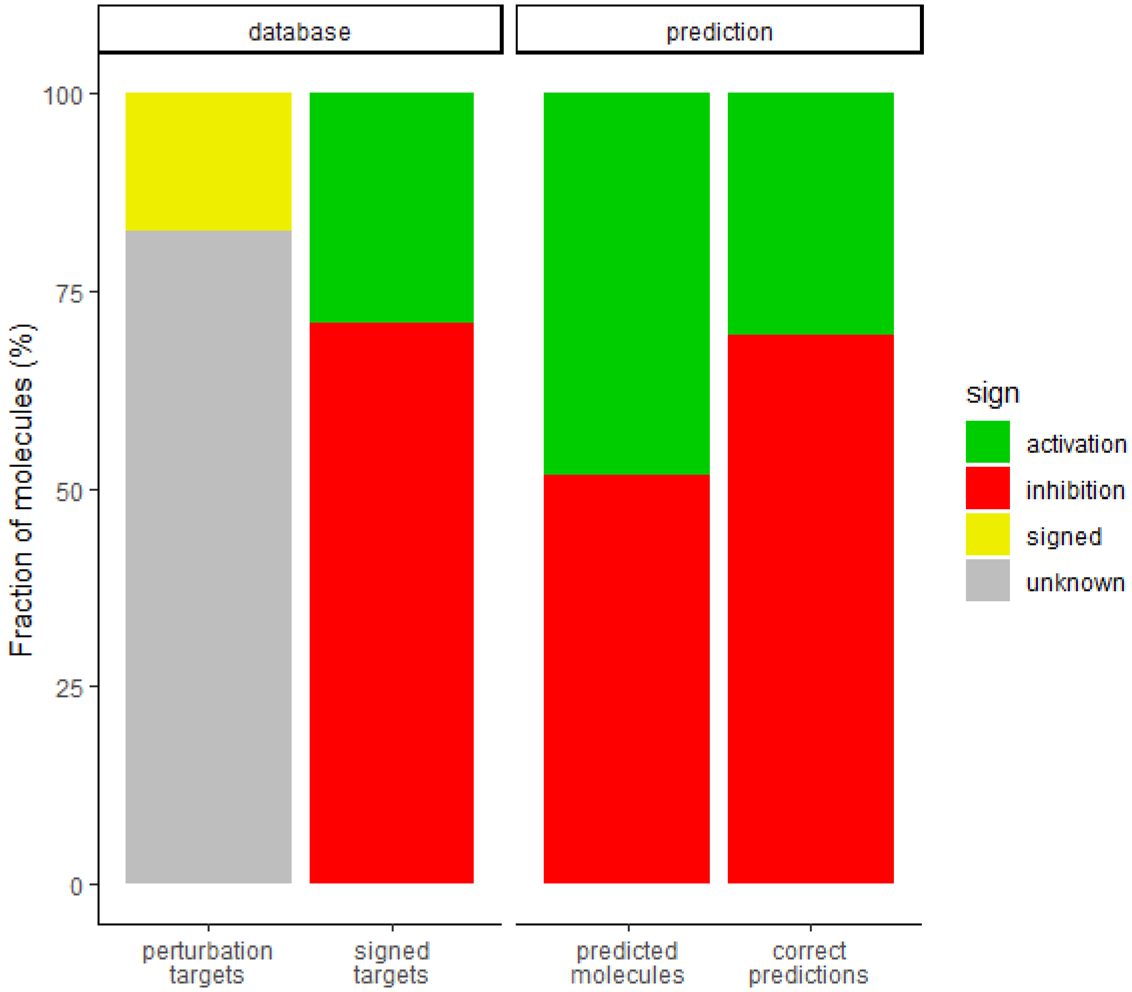

Figure S 7 Sign of perturbagens effect on their protein targets. The 84% of the drug-target pairs present in our datasets and contained in our signalling network (“perturbation targets”) is unsigned across STITCH, DrugBank and MetaCore. Of the known interactions (“signed targets”), 71% are inhibitory. In the predictions obtained with our method, 52% of the predicted signalling molecules are inhibited (“predicted molecules”). The correct predictions (true positives) are in majority inhibitions (69%).

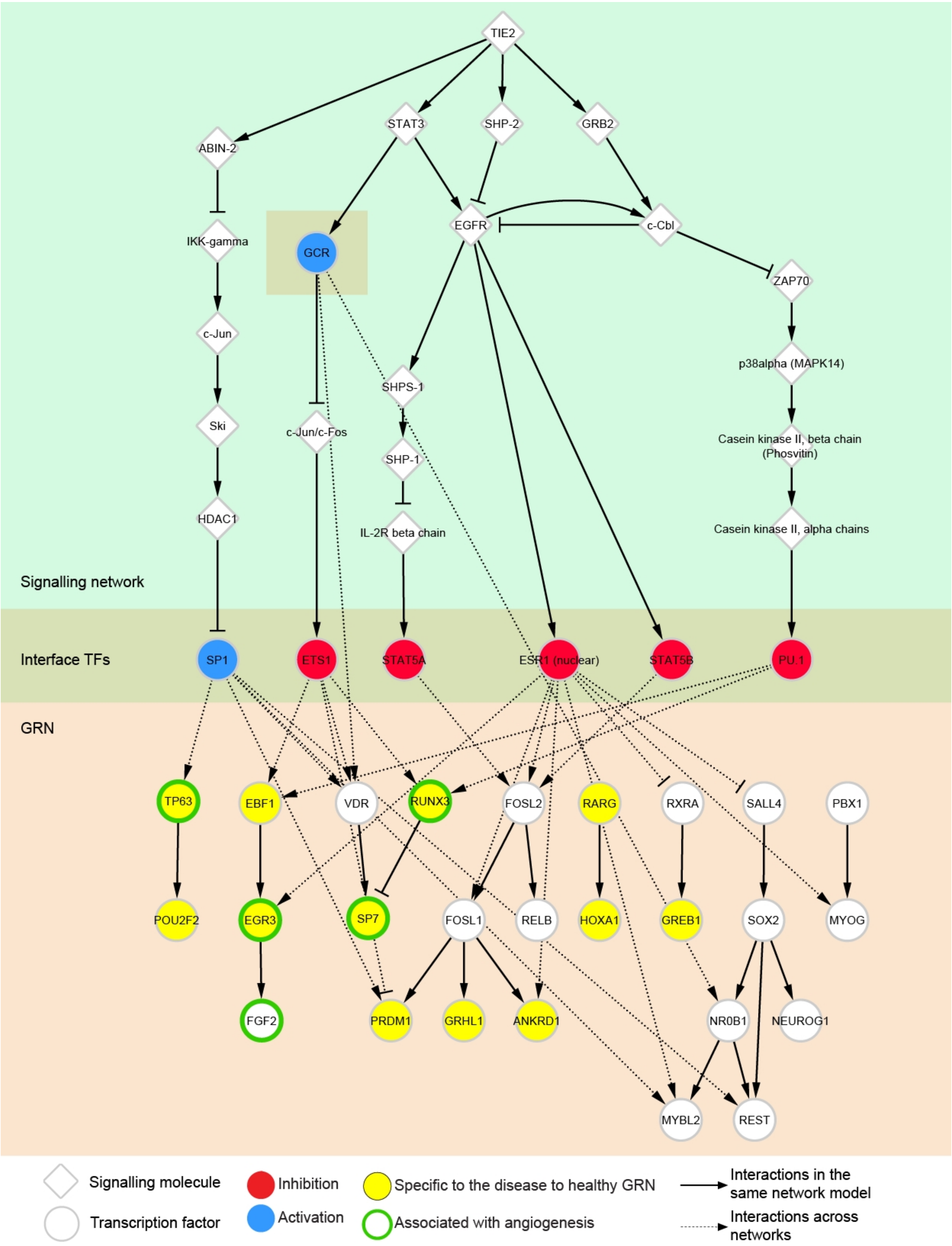

TIE2
ABIN-2
STAT3
SHP-2
GRB2
IKK-gamma
GCR
EGFR
c-Cbl
ZAP70
c-Jun
SHPS-1
p38alpha (MAPK14)
Ski
c-Jun/c-Fos
SHP-1
Casein kinase II, beta chain (Phosvitin)
HDAC1
IL-2R beta chain
Casein kinase II, alpha chains
Signalling network
Interface TFs
SP1
ETS1
STAT5A
ESR1 (nuclear)
STAT5B
PU.1
GRN
TP63
EBF1
VDR
RUNX3
FOSL2
RARG
RXRA
SALL4
PBX1
POU2F2
EGR3
SP7
FOSL1
RELB
HOXA1
GREB1
SOX2
MYOG
FGF2
PRDM1
GRHL1
ANKRD1
NR0B1
NEUROG1
MYBL2
REST
Signalling molecule
Transcription factor
Inhibition
Activation
Specific to the disease to healthy GRN
Associated with angiogenesis
Interactions in the same network model
Interactions across networks

Figure S 8 Integrated signalling and gene regulatory network for cirrhosis. The application of CVX-060 activates Tie2, which in turn activates (blue circles) or inhibits (red circles) interface TFs following the MPPs depicted. The interface TFs then act on the GRN representing the transition from disease to healthy state (disease GRN). In the GRN, white TFs are common between this GRN and the treated-to-healthy GRN, and yellow TFs are specific for the disease GRN. TFs that are associated aawith the regulation of angiogenesis are depicted with green border.
